# A multistart tabu search-based method for feature selection in medical applications

**DOI:** 10.1038/s41598-023-44437-4

**Published:** 2023-10-10

**Authors:** Joaquín Pacheco, Olalla Saiz, Silvia Casado, Silvia Ubillos

**Affiliations:** University of Burgos, Burgos, Spain

**Keywords:** Diseases, Mathematics and computing

## Abstract

In the design of classification models, irrelevant or noisy features are often generated. In some cases, there may even be negative interactions among features. These weaknesses can degrade the performance of the models. Feature selection is a task that searches for a small subset of relevant features from the original set that generate the most efficient models possible. In addition to improving the efficiency of the models, feature selection confers other advantages, such as greater ease in the generation of the necessary data as well as clearer and more interpretable models. In the case of medical applications, feature selection may help to distinguish which characteristics, habits, and factors have the greatest impact on the onset of diseases. However, feature selection is a complex task due to the large number of possible solutions. In the last few years, methods based on different metaheuristic strategies, mainly evolutionary algorithms, have been proposed. The motivation of this work is to develop a method that outperforms previous methods, with the benefits that this implies especially in the medical field. More precisely, the present study proposes a simple method based on tabu search and multistart techniques. The proposed method was analyzed and compared to other methods by testing their performance on several medical databases. Specifically, eight databases belong to the well-known repository of the University of California in Irvine and one of our own design were used. In these computational tests, the proposed method outperformed other recent methods as gauged by various metrics and classifiers. The analyses were accompanied by statistical tests, the results of which showed that the superiority of our method is significant and therefore strengthened these conclusions. In short, the contribution of this work is the development of a method that, on the one hand, is based on different strategies than those used in recent methods, and on the other hand, improves the performance of these methods.

## Introduction

The introduction is organized into 4 sub-sections: “Motivation”, “Literature on feature selection methods”, “Literature on feature selection in medicine”, and “Contribution”.

### Motivation

Diagnosis is a process in which a disease, injury, or other adverse effect is identified based on its signs and symptoms. To aid the establishment of a diagnosis, the clinician may use the patient’s medical history or conduct a physical examination and various tests, such as blood tests, imaging tests, and biopsies. In most pathologies, diagnosis has evolved toward faster and more accurate processes^1^.

In medicine, early diagnosis is vital for the effective treatment of some pathologies. For example, early detection improves patient survival in pathologies such as pancreatic cancer^2^. Moreover, early diagnosis is crucial to improve the prognosis of many diseases, e.g., tumors and melanomas^3^. In other diseases, such as diabetes, early diagnosis has become essential to alleviate the secondary complications of the disease, e.g., arterial lesions, hypercholesterolemia, hypertension, obesity, and myocardial infarction^4^. The diagnosis of Alzheimer’s disease in its earliest stages is very important, as it allows this disease to be differentiated from other neurodegenerative disorders with dementia^5^.

Data mining^6,7^ is a set of tools that have enabled the analysis of large amounts of information, generating patterns and norms that help to understand the behavior of a system. Within this set of tools, those used in prediction—and especially classification—have been adapted to the diagnosis of diseases. Their use in this scope has become an important emerging line of research^8^. With different models, such as those based on logistic regression^9^, discriminant analysis^10^, support vector machine^11^, neural networks^12^, classification trees^13^, nearest neighbor search^14^, and Bayesian classifiers^15^, among other approaches, it is possible to establish an early diagnosis.

Furthermore, in addition to good diagnostic accuracy, the models must be able to identify the most influential variables and features—that is, the ones that are truly relevant for diagnosis. In other words, it is useful to know which set of features optimize precision and other performance parameters in a diagnostic task. In previous works, the goal has been to determine which set of features, among the original ones, performs the diagnostic task in an optimal way ("optimal set").

In practice, this optimal set of features is generally unknown a priori, and the initial model usually contains irrelevant or redundant features^16^. Identifying the most relevant set of features and separating them from the redundant and/or irrelevant features is critical for successful diagnosis^17^. Therefore, it is important to incorporate feature selection in model creation to ensure the generation of simpler and more understandable models. Being able to reduce the complexity of a model may help to improve its precision and performance^18^ as well as the economy and speed with which data can be acquired and prepared^19,20^.

### Literature on feature selection methods

Feature selection methods can be classified into three types: filter, wrapper, and embedded^19^. Filter methods select a certain number of variables based on criteria such as correlation, likelihood, and information gain without the intervention of a classifier^19,20^. Some examples of filter methods are the following algorithms: correlation feature selection (or the correlation algorithm)^6^, mutual information^16^, the ReliefF algorithm^7^, the chi-square algorithm^21^, the Fisher score algorithm^22^, and the fast correlation-based filter^23^. Additionally, Hancer^24^ proposed a filter method based on differential evolution.

Wrapper methods explore different combinations or subsets of variables to evaluate the usefulness of each of these subsets through the predictive efficacy of a specific classifier. The aim is to find the best of these subsets. Wrapper methods usually generate better results than filter methods, since the latter do not evaluate the performance of the selected variables with a certain classifier^17^, although wrapper methods require longer computation times than filter methods. Due to the robustness of metaheuristic techniques in several complex applications, some of these techniques have been used to create wrapper methods for feature selection, such as genetic algorithms^25–27^, the gray wolf optimizer^28,29^, the flower pollination algorithm^30,31^, the bat algorithm^32,33^, the ant colony optimization algorithm^34,35^, the whale optimization algorithm^36,37^, particle swarm optimization^38,39^, the harmony search algorithm^40,41^, and the Harris hawk optimization algorithm^18,42^.

Finally, embedded methods integrate feature selection and classifier learning in a single process. These methods have been used in studies as^43–46^, and^47^.

### Literature on feature selection in medicine

Different approaches have been used to select variables/features and obtain models that establish diagnoses with high precision^48^. For example^49^, extracted different features of magnetic resonance imaging (MRI) data to select the most important characteristics for the diagnosis of brain tumors. Additionally, from MRI data^50^, defined biomarkers by quantifying the precision of different sets of morphological features to diagnose Alzheimer’s disease. Liu e al.^51^ developed a method to measure the performance of both individual features and different subsets of features to identify lung diseases. Similarly, Chong et al.^52^ created a classifier that enabled the detection of fibrotic interstitial lung disease using 3D texture features of computed tomography images. Shi et al.^53^ used a method based on feature selection that incorporates the clinician’s knowledge to achieve an accurate diagnosis of prostate problems. Additionally, Guinin et al.^54^ developed an automatic diagnostic tool for prostate pathology, and Sahran et al.^55^ proposed a new feature selection method from prostate histopathological images. Furthermore, feature selection studies have been conducted for different types of cancers. Thus, Jain et al.^56^ proposed a model to improve the diagnosis and the identification of cancer types. Wang et al.^57^ used a feature selection strategy for the diagnosis and classification of cancer with different gene expression profiles. Peng et al.^58^ used a forward feature selection algorithm for tumor classification. Finally, Kang et al.^59^ combined feature selection, logistic regression models, and support vector regression for tumor classification. Feature selection algorithms have also been combined with the AdaBoost classifier for the detection of glaucoma^60^.

An interesting and recent line of work in this field is that of methods based on evolutionary strategies inspired by biological metaphors. Thus, in Awadallah et al.^61,62^, strategies based on Rat Swarm and Horse Herd behavior were used. In order to improve these methods, different operators are analyzed. On the other hand, Braik et al.^63^ proposed three methods based on the Capuchin Search Algorithm, considering the k-Nearest Neighbor (k-NN) classifier.

One interesting field of application of feature selection in bio-medicine is gene interaction detection, such as single nucleotide polymorphisms (SNPs) epistatic interaction. In many cases, different metaheuristic strategies have been used. Thus, in the works of Tuo et al.^64,65^ this problem was approached from a multi-objective and multi-task optimization point of view. In both studies, methods based on harmony search optimization were proposed. In^66^ a similar approach was taken and a method based on ant colony optimization was proposed. Shang et al.^67^ proposed a simulation method combined with resampling methods.

In this paper, we propose a method based on tabu search and multi-start strategies. This is a stark difference with recent work based on evolutionary methods (such as those mentioned above). Evolutionary methods are, in general, more intuitive and easier to adapt and implement. Methods based on local search, such as the one proposed in this work, require greater adaptation to each specific problem and a greater implementation effort. On the other hand, our experience in different fields^68–70^ demonstrates that these local-search based methods (and specifically those based on tabu search) perform better than evolutionary methods. Indeed, as will be seen below, different computational tests show that our proposed method outperformed other recent methods as gauged by various metrics and classifiers.

### Contribution

The present study proposes a wrapper method of variable/feature selection that combines two metaheuristic strategies of combinatorial optimization: tabu search (TS) and multistart. Computational tests including several different classifiers were carried out with different diagnostic databases. These tests showed that our tool outperformed other known wrapper methods in the recent literature in terms of different metrics and classifiers. Our method obtains more efficient models—i.e. a better balance between the number of variables selected in the model and its precision. The results of various statistical tests support these conclusions.

The rest of the study is organized in the following manner: Section “Notation and formulation of the research problem” formulates the research problem formally; Section “Solution method for the proposed problem” describes the proposed method in detail; Section “Computational tests” describes the computational tests conducted to analyze the performance of our method against the other methods; and finally, Section “Conclusions” presents the conclusions.

## Notation and formulation of the research problem

To formulate the research problem that is addressed in this study (selection of variables for diagnosis), the data used to select the variables and to generate the models were defined as the training set. This dataset is $X$. The number of individuals/cases of *X* is *n*. The number of variables is $m$. Finally, the set of variables is $V$, and the *j*th variable is ${v}_{j}$, that is:

$$V=\left\{{v}_{1},{v}_{2},v,\dots ,{v}_{j},\dots ,{v}_{m}\right\}$$

For each individual of $X$, both the value of his or her features and his or her class (the presence or absence of the disease) are known. The problem lies in finding the $S\subset V$ subset that maximizes the objective function $f(S)$. This function is defined in the following manner:

$$f\left(S\right)=\beta \cdot Rat(S)+\left(1-\beta \right)\cdot (1-\frac{\left|S\right|}{m})$$

where $Rat(S)$ is defined as the rate of cases of $X$ that are well classified by the model obtained with the variables of *S* and the classifier considered. Parameter $\beta \in [\mathrm{0,1}]$ controls for the rate of correct diagnoses and the size of $\left|S\right|$. Therefore, the objective function $f$ balances the classifying capacity with the size of the set obtained. This objective function has been used in similar studies, such as^26,29,71^, and^38^. Usually, the $\beta$ values are equal or approximately equal to 0.99, which is the value we used in the present study.

## Solution method for the proposed problem

The solution method is a procedure (*MultiStartTabu*) that combines the multistart and TS strategies. In each iteration, two procedures are performed: a constructive procedure (*Constructive*) that generates an initial solution, and a second procedure that improves the solution generated by the constructive procedure. This improvement procedure (*TabuSearch*) is based on the TS strategy. The method ends when the solution does not improve after a series of iterations. Pseudocode 1 presents the $MultiStartTabu$ method.

**Figure:**
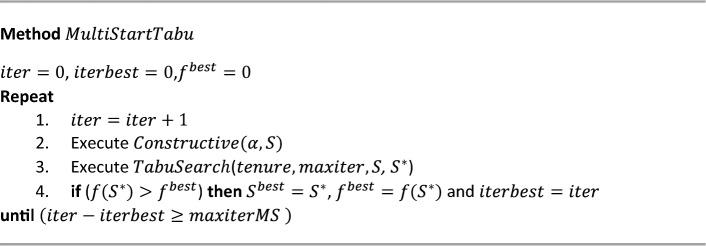
**Pseudocode 1.** $$MultiStartTabu$$ method.

As shown in the pseudocode, $iter$ is an auxiliary variable that indicates the number of the current iteration; $iterbest$ indicates where the best solution was found; ${S}^{best}$ and ${f}^{best}$ are the best solution and its classifying capacity, respectively; finally, $maxiterMS$ is a previously defined parameter indicating the number of iterations that must take place, with no improvement of ${f}^{best}$, in order for the method to conclude.

Next, we explain the $Constructive$ and $TabuSearch$ procedures. The $Constructive$ procedure begins with $S=\varnothing$; in each step, it selects an element of ${v}_{{j}^{*}}\in V-S$ among those that would best improve the objective function if added to *S*, and it is then added to *S*. The process ends when there are no elements in $V-S$ that improve the objective function $f$. Pseudocode 2 shows the $Constructive$ procedure.

**Figure:**
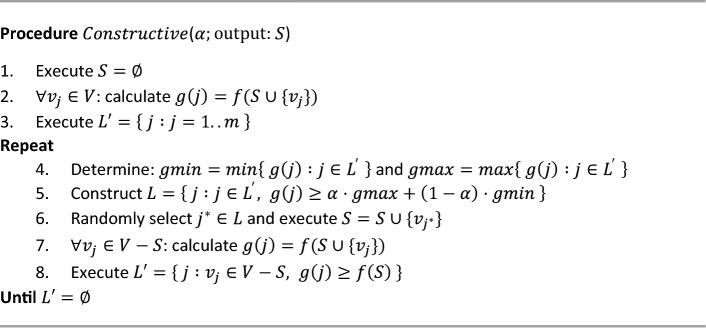
**Pseudocode 2**. $$Constructive$$ procedure.

The procedure is relatively simple. Initially, we execute $S=\varnothing$; in the following steps, for the ${v}_{j}$ elements that are not in $S$, the value of the objective function of *S* is calculated if ${v}_{j}$ is added ($g\left(j\right)=f(S\cup \left\{{v}_{j}\right\}$); then, the list *L*′ is created with the indices of the elements that improve the objective function of the current solution ($g\left(j\right)\ge f\left(S\right)$). Next, from the elements of the list *L*′, another list ($L$, a list of candidates) is created from the indices with the highest $g\left(j\right)$ values, and one of them is randomly selected (${j}^{*}$). Finally, the corresponding variable ${v}_{{j}^{*}}$ is added to $S$. The process ends when *L*′ = ∅ (there are no elements that improve the current solution). As can be observed, *L*′ is initially composed of all the indices of the elements of $V$ (considering $f\left(\varnothing \right)=0$). Parameter $\alpha$ regulates the size of $L$. It takes values between 0 and 1; thus, if $\alpha =0$, then $L=\left\{ j :{v}_{j}\in V-S\right\}$, and the process is completely random; on the other hand, if $\alpha =1$, then $L$ consists exclusively of index $j$, which corresponds to $gmax$, and the process is therefore deterministic. It is important to select an adequate value of $\alpha$ that allows different high-quality solutions to be obtained.

TS^72^ is a metaheuristic strategy that, in its basic version, consists of a neighbor search procedure. Each step analyses all the possible movements that can be made from the current solution, and the best movement is selected. Simple movements are used in order to ensure that each movement results in a solution that is relatively similar to the current solution (a nearby or “neighboring” solution). The procedure allows movements to solutions that do not improve the current solution. Moreover, to prevent the algorithm from cycling, some movements are declared “tabu” and are initially not considered.

In our case, we considered three types of movements: a) adding an element ${v}_{{j}{\prime}}\in V-S$; b) removing an element ${v}_{j}\in S$; and c) exchanging an element ${v}_{j}\in S$ with another element ${v}_{{j}{\prime}}\in V-S.$ The set of neighbor solutions of $S$ (i.e., those that are reached through these movements) is defined as $N(S)$.

To avoid cycles, the output from *S* (throughout a series of iterations) of elements that recently entered *S* is declared “tabu”. Similarly, the input into *S* of elements that recently left *S* is also declared “tabu”. To verify the tabu status of the entry or exit of an element ${v}_{j}\in V$, we defined:

$VectorIn\left(j\right)$:Number of the iteration in which the element ${v}_{j}$ entered $S$.

$VectorOut\left(j\right)$:Number of the iteration in which the element ${v}_{j}$ left $S$.

Thus, the entry of an element ${v}_{{j}{\prime}}\in V-S$ is tabu if

1 $$iter\le VectorOut\left({j}{\prime}\right)+tenure$$

Additionally, the exit of an element ${v}_{j}\in S$ is tabu if

2 $$iter\le VectorIn\left(j\right)+tenure$$

Finally, the exchange of an element ${v}_{j}\in S$ with an element ${v}_{{j}{\prime}}\in V-S$ is tabu if either of the two aforementioned conditions is confirmed to be present.

The parameter $tenure$ indicates the number of iterations in which an output or input is tabu. The auxiliary variable $iter$ represents the number of iterations. On the other hand, the tabu status of a movement can be ignored (and thus the movement can be considered) if such movement results in a solution with a greater value of the objective function $f$ than the previous solutions visited (“aspiration criterion”). Pseudocode 3 shows the $TabuSearch$ procedure.

**Figure:**
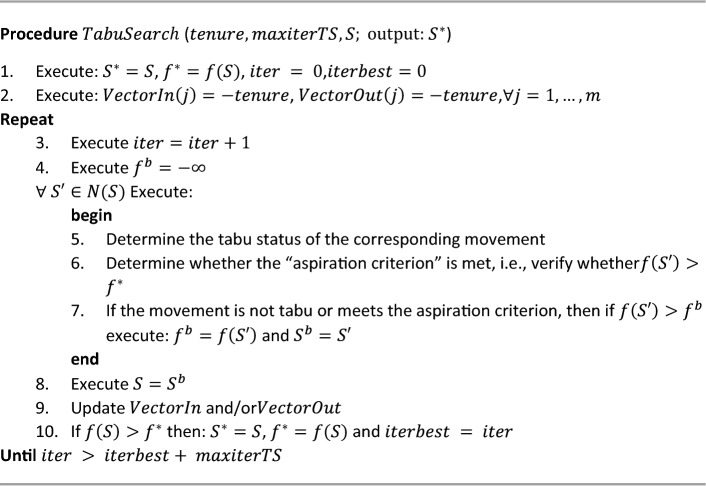
**Pseudocode 3**. $$TabuSearch$$ procedure.

As can be observed in Pseudocode 3, each iteration considers all the movements that are not tabu or that meet the aspiration criterion. The best neighbor solution considered is stored in the variable ${S}^{b}$. This change is executed (${f}^{b}=f\left(S{\prime}\right) \mathrm{and} S{=S}^{b}$), and the values of $VectorIn$ and/or $VectorOut$ are updated according to the type of movement performed and the elements involved. After each iteration, ${S}^{*}$ and ${f}^{*}$, which are the best solution found during the search and its value of the objective function $f$, respectively, are updated. The procedure ends after a preestablished number of iterations ($maxiterTS$) have taken place with no improvement of ${f}^{*}$. In this procedure, the parameter $tenure$ plays an important role: high values result in many movements being declared tabu, thus reducing the flexibility of the process; low values may not prevent cycles. Therefore, adequate selection is critical.

## Computational tests

This section describes different computational tests. The first group of tests was performed to adjust the parameters of the proposed model (Section “Fine-tuning of parameters”). The second group of tests was conducted to compare our method with other popular variable selection methods in the recent literature (Section “Comparison with other methods”). As can be observed in Section “Comparison with other methods”, our method, in general, obtained better results according to several metrics. Multiple databases of medical diagnoses were used.

To carry out these tests, nine databases were used: eight databases from the well-known repository of the University of California, Irvine (USA) (https://archive.ics.uci.edu/) and another database of Alzheimer’s diagnoses, which is presented for the first time in this study (https://www.ubu.es/metaheuristicos-grinubumet/ejemplos-y-datos-de-problemas). Because these databases concern diagnoses, the data were divided into two different types (presence of the disease, or “positive,” and absence of the disease, or “negative”). Table 1 shows descriptions of the different databases and their characteristics.

**Table 1:** Databases used.

Database name	Cases (pos/neg)	Features
Parkinson	195 (46/146)	22
Quality assessment of digital colposcopies (QADC)	287 (72/215)	67
SPECTF Heart	267 (56/211)	44
Wisconsin breast cancer—diagnosis (WDBC)	569 (212/357)	30
Wisconsin breast cancer—prognosis (WPBC)	198 (151/47)	31
Alzheimer	1548 (630/918)	28
Cervical cancer	668 (605/63)	28
Glioma	838 (486/352)	23
Mesothelioma	324 (228/96)	33

It should be noted that in the Cervical database, the two variables with the highest number of missing values were initially eliminated, and then the cases with missing values were eliminated.

### Fine-tuning of parameters

To adjust the parameters, three of the nine databases were considered: one with few features (*Parkinson*), another with many features (Quality Assessment of Digital Colposcopies, or *QADC*), and another with an intermediate number of features (Wisconsin Breast Cancer – Prognosis, or *WPBC*). Discriminant analysis was used as a classifier due to its rapid calculation capacity.

**Figure 1:**
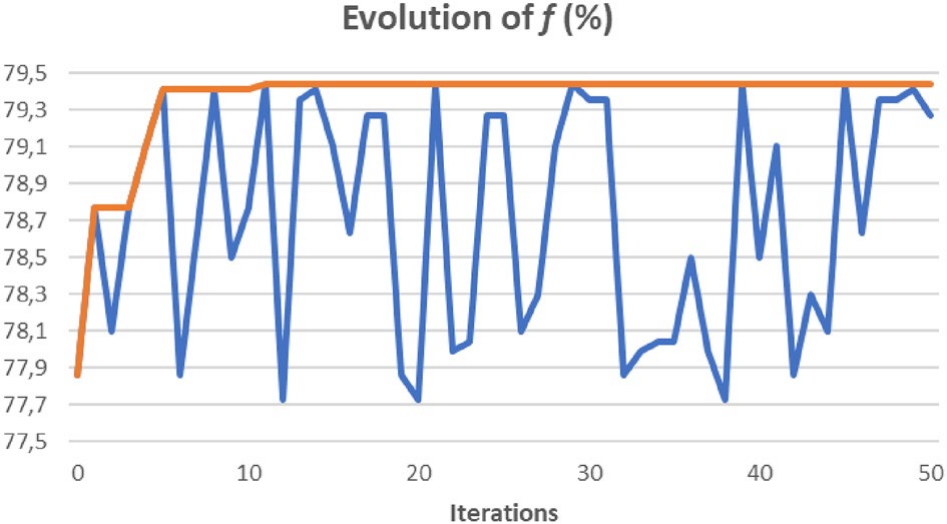
Evolution of the value of $f$ for *WPBC.*

Four parameters in our $MultiStartTabu$ (MST) method were analysed: $\alpha$, $tenure, maxiterTS$ and $maxiterMS$. The parameter $maxiterTS$ was used as the stopping criterion in the $TabuSearch$ procedure, whereas $maxiterMS$ was used as the stopping criterion in the general MST procedure. To analyze the parameters $\alpha$ and $tenure$, we set the values $maxiterTS=10\cdot n$ and $maxiterMS=20$. For $\alpha$, we considered the following values: $\alpha$ = 0, 0.1, 0.5, 0.9, 0.99, and 1. For $tenure$, we considered the following values: $tenure=n/2,n, 2\cdot n$ and $5\cdot n$. After analyzing the 20 combinations, we found the best results at $\alpha$ = 0.99 and $tenure=n/2$. Subsequently, the parameters $maxiterTS$ and $maxiterMS$ were analyzed using these values for $\alpha$ and $tenure$. It was observed that with values higher than $maxiterTS=10\cdot n$ and $maxiterMS=10$, there were no significant improvements. Therefore, we selected these values. Figure 1 below shows the evolution of the objective function $f$ over a series of iterations that make up the $MultiStartTabu$ method for the WPBC database. The blue line indicates the values obtained in each iteration, and the red line shows the evolution of the best value. It can be seen that with a relatively low number of iterations the best final value is already reached.

### Comparison with other methods

#### Baseline methods, classifiers, metrics, and experimental details

This subsection describes the tests and the corresponding results that were used to compare our method with five recent wrapper methods (baseline methods): *genetic algorithm*, or GA^26^; *gray wolf optimizer*, or GWO^71^; *particle swarm optimization*, or PSO^39^; *whale optimization algorithm*, or WOA^36^; and *flower pollination algorithm*, or FPA^31^.

As classifiers, we used discriminant analysis (DA), logistic regression (LR), and a support vector machine (SVM). For the experiments, we used a k-fold cross-validation design ($k=10$), which allowed us to conduct statistical tests. In addition to the value of the objective function, the following metrics were used:

$$ACC (Accuracy)=\frac{TP+TN}{TP+FP+TN+FN}$$

$$AUC (Area \, Under \, the \, Curve)=\left(1+\frac{TP}{TP+FN}-\frac{FP}{TN+FP}\right)/2$$

$$Gmean \left(Geometric \, Mean\right)=\sqrt{\frac{TP}{TP+FN}\cdot \frac{TN}{TN+FP}}$$

$$F1 \left(F1 \, Score\right)=\frac{2\cdot TP}{2\cdot TP+FP+FN}$$

where $TP$, $TN$, $FP$, and $FN$ represent true positive, true negative, false positive, and false negative results, respectively. As can be observed, the definition of $ACC$ is equivalent to that of $Rat$, although it refers to the training set. The three metrics are also frequently employed and very useful when the subsets of each class are unbalanced or poorly balanced.

For each database, the following pre-processing steps were carried out: the data of the features were normalized, and the database was divided into 10 folds, resulting in 10 training set–test set pairs. Subsequently, for each database and classifier, the following process was performed:

- For each training set, our MST algorithm was executed with the previously considered parameters, and the computation time was recorded. Then, each of the other 5 methods was executed, with the computation time of the MST as the stopping criterion.
- The results obtained for each fold of each of the methods were recorded (objective function on the training set, metrics on the validation set, and mean difference tests).
- All algorithms were implemented with the Object-Pascal programming language using the Delphi compiler and the Rad Studio development environment (version 10.3). All tests were performed on a computer with an i9-10920X processing unit and 128 GB RAM. The parameters used by the baseline methods were the ones recommended by the corresponding articles, except for the stopping criterion, as was commented on above.
- For the estimation of the logistic regression models, we used the algorithm of Lin et al.^73^, whereas for the SVM models, we used the method proposed by Hsieh et al.^74^.

Table 2 shows the computation times used by our MST (and thus by the rest of the methods) for each of the databases and classifiers. The mean time and standard deviation on the set of the 10 folds are indicated.

**Table 2:** Computation time in seconds (mean and standard deviation) for each method.

	DA	LR	SVM
Parkinson	0.529 ± 0.033	17.761 ± 1.095	10.412 ± 0.690
QADC	71.748 ± 6.488	1906.915 ± 209.946	1513.337 ± 177.693
SPECTF heart	9.091 ± 0.620	642.467 ± 80.625	383.022 ± 47.990
WDBC	1.187 ± 0.020	171.111 ± 13.576	176.336 ± 13.383
WPBC	1.019 ± 0.045	86.006 ± 11.781	37.534 ± 1.407
Alzheimer	1.630 ± 0.089	260.755 ± 37.853	274.872 ± 35.308
Cervical cancer	1.386 ± 0.021	392.119 ± 23.511	227.206 ± 24.201
Glioma	1.463 ± 0.062	162.417 ± 10.001	156.452 ± 11.901
Mesothelioma	3.125 ± 0.099	763.716 ± 71.011	396.045 ± 42.541

As can be observed, the computation times were longer in the databases with a larger number of variables (*QADC*) than in those with a smaller number of variables (*Parkinson*). Moreover, for the calculation of the objective function $f(S)$ and, more specifically, the rate of correct diagnoses $Rat(S)$, it was necessary to generate the model with the corresponding classifier using the variables of $S$. In the case of DA, the models were obtained easily and immediately, whereas in the case of LR and SVM, the models were obtained through a more complex optimization process, as mentioned above.

#### Results of DA

Next, we show the results obtained for each classifier. First, the results obtained for DA are presented. Table 3 displays the results obtained for the objective function $f$, with the mean and standard deviation for each database and method. The method with the best mean is indicated in bold text. From these results, a paired two-tailed t test was carried out with each of the other methods. Significant differences in favor of our method are indicated with “+” after the result of the method that it is compared with; negative differences in favor of the other method are indicated with “−”; and “=” represents the absence of significant differences. The last row (W/T/L) indicates, for each baseline method, the number of “wins” (W) (i.e., the number of significant differences in favor of our method compared to the other method), “ties” (T) and “losses” (L).

**Table 3:** Results of $f$ (%) using DA.

	MST	GA	GWO	PSO	WOA	FPA
Parkinson	**83.76 ± 0.36**	83.04 ± 0.43 (+)	83.31 ± 0.24 (+)	82.69 ± 0.40 (+)	82.56 ± 0.72 (+)	82.84 ± 0.54 (+)
QADC	**83.95 ± 0.51**	82.96 ± 0.23 (+)	83.25 ± 0.50 (+)	82.68 ± 0.41 (+)	82.27 ± 0.30 (+)	82.60 ± 0.40 (+)
SPECTF Heart	**81.49 ± 0.52**	80.53 ± 0.37 (+)	80.94 ± 0.50 (+)	80.15 ± 0.56 (+)	79.63 ± 0.45 (+)	80.01 ± 0.52 (+)
WDBC	**95.58 ± 0.16**	95.21 ± 0.32 (+)	95.35 ± 0.15 (+)	94.98 ± 0.26 (+)	94.86 ± 0.44 (+)	95.21 ± 0.32 (+)
WPBC	**79.44 ± 0.48**	78.50 ± 0.23 (+)	78.77 ± 0.42 (+)	78.10 ± 0.47 (+)	77.73 ± 0.35 (+)	77.99 ± 0.40 (+)
Alzheimer	**82.38 ± 0.45**	81.73 ± 0.46 (+)	81.83 ± 0.32 (+)	81.35 ± 0.33 (+)	81.41 ± 0.58 (+)	81.72 ± 0.47 (+)
Cervical Cancer	**91.53 ± 0.19**	91.27 ± 0.30 (+)	91.26 ± 0.31 (+)	90.87 ± 0.43 (+)	90.65 ± 0.45 (+)	91.08 ± 0.31 (+)
Glioma	**89.56 ± 0.70**	88.18 ± 00.51 (+)	88.60 ± 0.67 (+)	87.74 ± 0.54 (+)	87.51 ± 0.77 (+)	87.96 ± 0.60 (+)
Mesothelioma	**75.03 ± 0.64**	74.16 ± 00.6 (+)	74.27 ± 0.56 (+)	73.72 ± 0.54 (+)	73.66 ± 0.82 (+)	74.00 ± 0.68 (+)
W/T/L		9/0/0	9/0/0	9/0/0	9/0/0	9/0/0

The best values are in bold.

**Figure 2:**
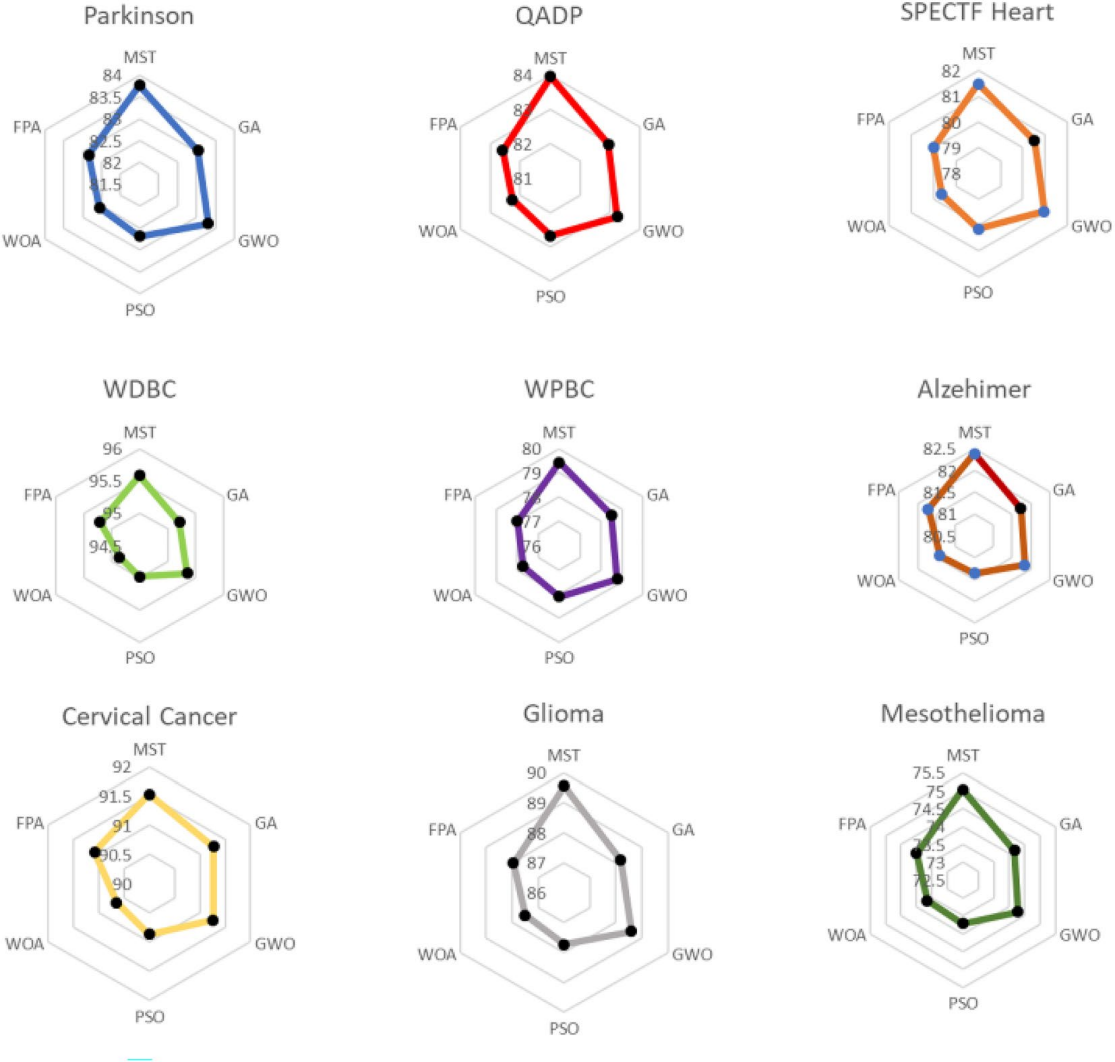
Radial plots of the mean values of the objective function $f$ (%) of the different methods using DA.

From Table 3, it can be observed that, in all databases, our method obtained the best results in terms of the objective function $f$. Moreover, in all cases, the difference with respect to each of the other methods was significant. Figure 2 shows nine radial plots (one per database) representing the mean results of the different methods.

**Figure 3:**
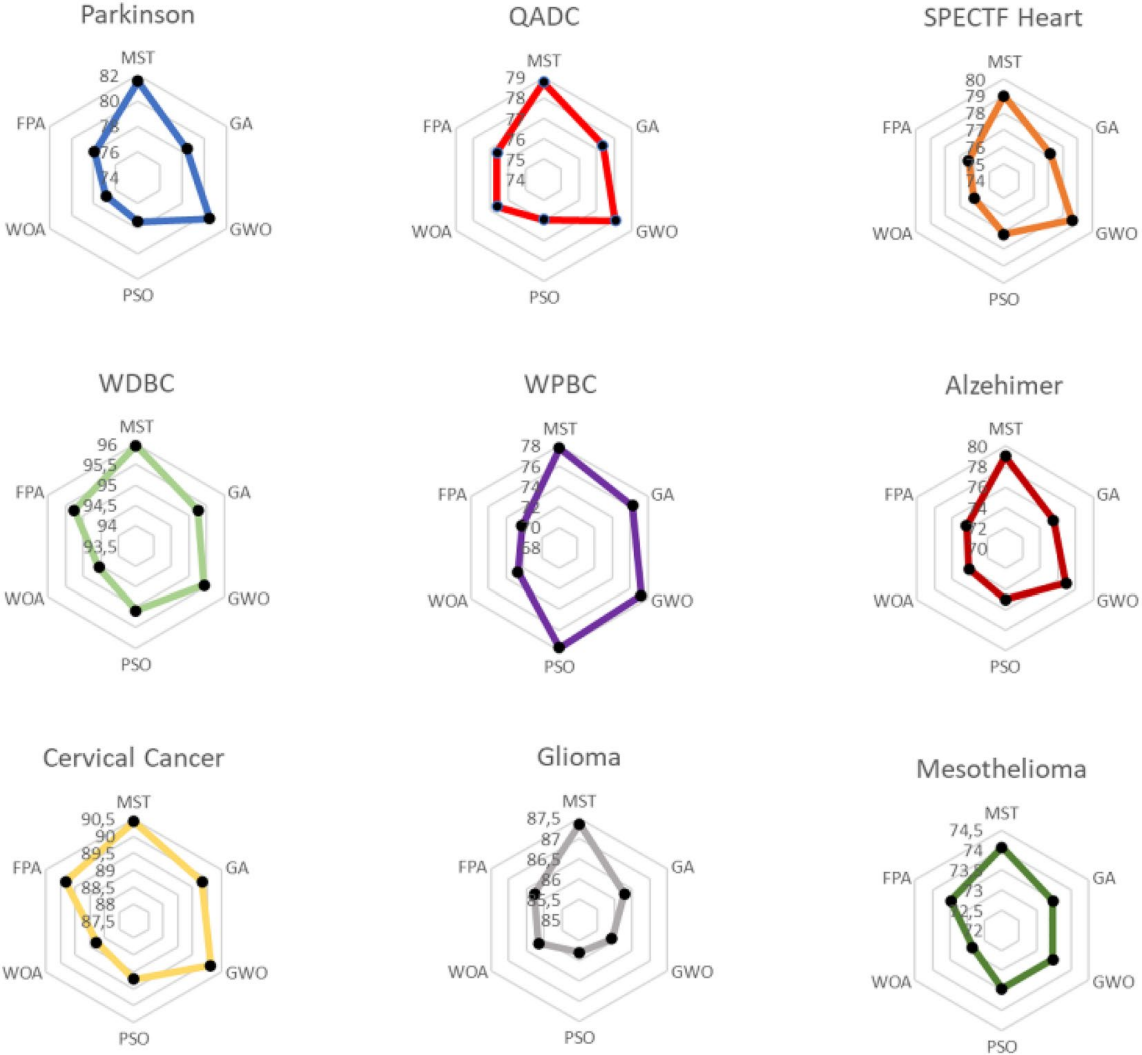
Radial plots of the mean $ACC$ values (%) of the different methods using DA.

Similarly, Table 4 and Fig. 3 present the results of the $ACC$ metric on the test set. Table 4 shows the means, standard deviations, and results of t tests (as in Table 3). Figure 3 displays radial plots of the mean results. From Table 4 and Fig. 3, the following can be observed:

- In all databases, our method obtained better mean results than any of the other methods tested. The PSO method obtained the same mean result only in the *WPBC* database.
- Moreover, in most cases (combinations of databases/baseline method), these differences were significant. Only in 10 of the 45 cases were the differences not significant. Specifically, in the t tests compared to GWO, our method “tied” in 5 databases and “won” in 4 of them. In general, the results obtained by the GWO method were relatively similar to those obtained by our MST method.

**Table 4:** Results of $ACC$ (%) using DA.

	MST	GA	GWO	PSO	WOA	FPA
Parkinson	**81.55 **$\pm 2$**.50**	78.45 $\pm$ 2.24 (+)	80.50 $\pm$ 2.23 (=)	77.47 $\pm$ 2.15 (+)	76.89 $\pm$ 2.90 (+)	77.95 $\pm$ 2.41 (+)
QADC	**78.76** $\pm$ **2.44**	77.34 $\pm$ 2.55 (+)	78.07 $\pm$ 2.69 (=)	75.96 $\pm$ 1.90 (+)	76.64 $\pm$ 2.49 (+)	76.65 $\pm$ 2.36 (+)
SPECTF Heart	**79.02** $\pm$ **2.01**	77.15 $\pm$ 1.15 (+)	78.63 $\pm$ 2.00 (=)	77.14 $\pm$ 1.47 (+)	76.03 $\pm$ 2.61 (+)	76.41 $\pm$ 2.41 (+)
WDBC	**95.96** $\pm$ **0.84**	95.26 $\pm$ 0.84 (+)	95.43 $\pm$ 0.90 (+)	95.08 $\pm$ 1.10 (+)	94.56 $\pm$ 1.28 (+)	95.26 $\pm$ 0.84 (+)
WPBC	**77.76** $\pm$ **3.59**	76.26 $\pm$ 3.36 (=)	77.26 $\pm$ 4.30 (=)	**77.76** $\pm$ **2.71** (=)	72.74 $\pm$ 4.80 (+)	72.24 $\pm 4$.80 (+)
Alzheimer	**79.01** $\pm$ **3.40**	75.45 $\pm$ 2.09 (+)	76.87 $\pm$ 3.27 (+)	75.00 $\pm$ 2.38 (+)	74.10 $\pm$ 0.90 (+)	74.35 $\pm$ 0.57 (+)
Cervical Cancer	**91.62 **$\pm 0$**.77**	91.17 $\pm 0$.83(+)	91.17 $\pm 1$.09 (+)	89.52 $\pm$ 1.00 (+)	89.37 $\pm$ 1.01 (+)	90.27 $\pm 1$.26 (+)
Glioma	**88.30 **$\pm 0$**.52**	87.35 $\pm 0$.83(+)	87.71 $\pm 0$.79 (+)	86.64 $\pm$ 0.74 (+)	86.28 $\pm$ 1.00 (+)	86.99 $\pm 0$.89 (+)
Mesothelioma	**74.07** $\pm$ **1.58**	73.47 $\pm$ 1.36 (=)	73.47 $\pm$ 1.36 (=)	73.47 $\pm$ 1.36 (=)	72.85 $\pm$ 2.27 (+)	73.47 $\pm$ 1.36 (=)
W/T/L		7/2/0	4/5/0	7/2/0	9/0/0	8/1/0

The best values are in bold.

Tables 5, 6, and 7 show the results of the $AUC$, $GMean$, and $F1$ metrics, respectively, as in the previous tables. The conclusions of these results are very similar to those drawn for the $ACC$ metric:

- Our MST model obtained a better mean result than the other methods in almost all cases. There was only one case in which the mean value of a baseline method was the same (the $AUC$ metric, *WPBC* database, and PSO method), and there were another two cases in which the result of a baseline method was slightly better (the $Gmean$ metric, *WPBC* database, and PSO method; the $Gmean$ metric, *QADC* database and GA method), although the differences were not significant.
- Moreover, in most cases, the differences in favor of our method were significant. Only the results of the GWO method were relatively similar to those of our method in terms of the three metrics, according to the t tests (5 not significant differences and 4 significant differences in favor of our method in $AUC$ and 6 not significant differences and 3 significant differences in favor of our method in $Gmean$ and $F1$).

**Table 5:** Results of $AUC$ (%) using DA.

	MST	GA	GWO	PSO	WOA	FPA
Parkinson	**80.85 ± 1.56**	78.77 ± 1.57 (+)	80.15 ± 1.73 (=)	78.13 ± 1.41 (+)	77.73 ± 1.90 (+)	78.44 ± 1.63 (+)
QADC	**64.15 ± 3.50**	64.10 ± 4.43 (=)	63.71 ± 3.94 (=)	61.33 ± 3.26 (+)	62.67 ± 4.49 (=)	62.76 ± 3.83 (=)
SPECTF Heart	**86.72 ± 1.31**	85.55 ± 0.51 (+)	86.48 ± 1.25 (=)	85.54 ± 0.80 (+)	84.85 ± 1.45 (+)	85.09 ± 1.37 (+)
WDBC	**94.86 ± 0.66**	94.22 ± 0.74 (+)	94.44 ± 0.73 (+)	94.07 ± 1.04 (+)	93.17 ± 1.61 (+)	94.22 ± 0.74 (+)
WPBC	**77.31 ± 3.59**	75.65 ± 4.29 (=)	76.31 ± 4.93 (=)	**77.31 ± 3.23** (=)	69.75 ± 7.50 (+)	68.50 ± 7.56 (+)
Alzheimer	**76.85 ± 3.67**	73.08 ± 2.30 (+)	74.60 ± 3.52 (+)	72.65 ± 2.49 (+)	71.66 ± 0.81 (+)	71.90 ± 0.47 (+)
Cervical Cancer	**57.07 ± 0.70**	55.99 ± 2.83 (+)	56.90 ± 0.72 (=)	55.66 ± 2.68 (+)	53.91 ± 3.59 (+)	55.99 ± 2.83 (+)
Glioma	**88.19 ± 1.47**	87.16 ± 1.05 (+)	86.77 ± 0.86 (+)	86.62 ± 1.10 (+)	87.05 ± 1.06 (+)	87.16 ± 1.05 (+)
Mesothelioma	**58.69 ± 0.88**	58.25 ± 0.59 (+)	58.25 ± 0.59 (+)	58.25 ± 0.59 (+)	57.81 ± 1.30 (+)	58.25 ± 0.59 (+)
W/T/L		7/2/0	4/5/0	8/1/0	8/1/0	8/1/0

The best values are in bold.

**Table 6:** Results of $Gmean$ (%) using DA.

	MST	GA	GWO	PSO	WOA	FPA
Parkinson	**80.81 ± 1.54**	78.75 ± 1.61 (+)	80.14 ± 1.71 (=)	78.10 ± 1.44 (+)	77.67 ± 1.95 (+)	78.41 ± 1.68 (+)
QADC	56.72 ± 5.98	**57.94 ± 7.02 (=)**	56.47 ± 6.23 (=)	53.60 ± 5.31 (+)	55.65 ± 7.08 (=)	55.90 ± 6.17 (=)
SPECTF Heart	**85.69 ± 1.52**	84.32 ± 0.61 (+)	85.41 ± 1.45 (=)	84.31 ± 0.96 (+)	83.47 ± 1.76 (+)	83.75 ± 1.66 (+)
WDBC	**94.76 ± 0.63**	94.12 ± 0.74 (+)	94.36 ± 0.71 (+)	93.99 ± 1.04 (+)	92.99 ± 1.71 (+)	94.12 ± 0.74 (+)
WPBC	77.20 ± 3.75	75.51 ± 4.46 (=)	76.18 ± 5.09 (=)	**77.22 ± 3.41** (=)	69.20 ± 8.16 (+)	67.83 ± 8.23 (+)
Alzheimer	**75.94 ± 3.93**	71.94 ± 2.50 (+)	73.57 ± 3.77 (+)	71.53 ± 2.63 (+)	70.45 ± 0.75 (+)	70.68 ± 0.39 (+)
Cervical Cancer	**39.55 ± 1.44**	35.44 ± 12.54 (+)	39.49 ± 1.47 (=)	35.30 ± 12.49 (+)	27.17 ± 18.80 (+)	35.44 ± 12.54 (+)
Glioma	**88.03 ± 1.49**	86.97 ± 1.09 (+)	86.60 ± 0.89 (+)	86.47 ± 1.13 (+)	86.86 ± 1.09 (+)	86.97 ± 1.09 (+)
Mesothelioma	**44.87 ± 1.13**	44.68 ± 1.23 (=)	44.68 ± 1.23 (=)	44.68 ± 1.23 (=)	44.47 ± 1.46 (=)	44.68 ± 1.23 (=)
W/T/L		6/3/0	3/6/0	7/2/0	7/2/0	7/2/0

The best values are in bold.

**Table 7:** Results of $F1$ (%) using DA.

	MST	GA	GWO	PSO	WOA	FPA
Parkinson	**86.92 ± 2.09**	84.39 ± 2.02 (+)	86.10 ± 1.76 (=)	83.58 ± 1.86 (+)	83.04 ± 2.66 (+)	83.97 ± 2.15 (+)
QADC	**86.82 ± 1.62**	85.72 ± 1.50 (+)	86.34 ± 1.69 (=)	84.97 ± 1.17 (+)	85.34 ± 1.45 (+)	85.34 ± 1.45 (+)
SPECTF Heart	**84.66 ± 1.73**	83.11 ± 0.71 (+)	84.35 ± 1.65 (=)	83.09 ± 1.12 (+)	82.11 ± 2.06 (+)	82.44 ± 1.95 (+)
WDBC	**96.85 ± 0.69**	96.29 ± 0.68 (+)	96.43 ± 0.72 (+)	96.15 ± 0.89 (+)	95.79 ± 0.96 (+)	96.29 ± 0.68 (+)
WPBC	**61.98 ± 5.56**	59.70 ± 5.32 (=)	60.82 ± 6.74 (=)	61.88 ± 4.79 (=)	52.37 ± 9.32 (+)	50.91 ± 10.05 (+)
Alzheimer	**71.62 ± 4.90**	66.64 ± 3.10 (+)	68.67 ± 4.68 (+)	66.13 ± 3.27 (+)	64.80 ± 0.93 (+)	65.09 ± 0.47 (+)
Cervical Cancer	**23.97 ± 2.13**	21.02 ± 7.84 (+)	23.39 ± 1.82 (=)	19.89 ± 7.22 (+)	14.16 ± 10.35 (+)	21.02 ± 7.84 (+)
Glioma	**86.14 ± 1.55**	85.01 ± 1.14 (+)	84.60 ± 0.91 (+)	84.45 ± 1.18 (+)	84.90 ± 1.12 (+)	85.01 ± 1.14 (+)
Mesothelioma	**32.31 ± 1.32**	31.79 ± 1.32 (=)	31.79 ± 1.32 (=)	31.79 ± 1.32 (=)	31.36 ± 1.90 (+)	31.79 ± 1.32 (=)
W/T/L		7/2/0	3/6/0	7/2/0	9/0/0	8/1/0

The best values are in bold.

Finally, Table 8 shows a summary of the test results (“wins”, “ties” and “losses”), in terms of both the objective function $f$ and each of the metrics, for each baseline method. The last row indicates the total sum of “wins”, “ties” and “losses” for each baseline method. From these results, it can be concluded that our method obtained better results than the rest of the methods. With respect to the objective function $f$, all differences were significant in favor of our method. Regarding the metrics, compared to GA, PSO, WOA, and FPA, significant differences were obtained in most cases (of 36 cases, there were 27 significant differences compared to GA, 29 compared to PSO, 33 compared to WOA and 31 compared to FPA). Compared to GWO alone, fewer significant differences were observed (13 out of 36). There were no significant differences in favor of any of the other methods.

**Table 8:** Summary of the t test results for $f$ and the different metrics with DA.

	GA	GWO	PSO	WOA	FPA
Objective function* f*	9/0/0	9/0/0	9/0/0	9/0/0	9/0/0
Metrics on test sets					
ACC	7/2/0	3/6/0	7/2/0	9/0/0	8/1/0
AUC	7/2/0	4/5/0	8/1/0	8/1/0	8/1/0
Gmean	6/3/0	3/6/0	7/2/0	7/2/0	7/2/0
F1	7/2/0	3/6/0	7/2/0	9/0/0	8/1/0
Total (W/T/L)	27/9/0	13/23/0	29/7/0	33/3/0	31/5/0

#### Results of LR

Next, we analyze the results obtained for LR. To avoid an excessive number of tables and figures, we present only Table 9, which shows the results of the accuracy ($ACC$) metric (means, standard deviations, and t tests), and Table 10, which displays a summary of the t tests (“Wins”, “Ties” and “Losses”), both for the objective function $f$ and for each of the metrics, as applied to each baseline method.

**Table 9:** Results of $ACC$ (%) using LR.

	MST	GA	GWO	PSO	WOA	FPA
Parkinson	**87.16 ± 3.66**	85.13 ± 1.58 (+)	85.13 ± 1.58 (+)	85.13 ± 1.58 (+)	85.13 ± 1.58 (+)	85.13 ± 1.58 (+)
QADC	**84.68 ± 2.33**	83.28 ± 2.17 (+)	84.34 ± 2.33 (=)	83.28 ± 2.17 (+)	81.54 ± 1.56 (+)	82.93 ± 2.51 (+)
SPECTF Heart	**87.29 ± 3.50**	85.80 ± 2.16 (+)	**87.29 ± 3.03 (=)**	85.80 ± 2.16 (+)	85.03 ± 1.66 (+)	84.64 ± 2.14 (+)
WDBC	**97.71 ± 1.68**	96.31 ± 1.55 (+)	96.31 ± 1.55 (+)	96.31 ± 1.55 (+)	95.43 ± 1.23 (+)	95.96 ± 1.45 (+)
WPBC	**86.39 ± 2.25**	85.39 ± 3.55 (=)	85.89 ± 3.02 (=)	84.89 ± 3.94 (=)	83.39 ± 3.93 (+)	83.39 ± 3.93 (+)
Alzheimer	**81.20 ± 2.15**	79.46 ± 1.22 (+)	79.97 ± 1.49 (+)	79.13 ± 1.25 (+)	78.29 ± 1.17 (+)	78.94 ± 0.99 (+)
Cervical Cancer	**91.17 ± 1.09**	90.12 ± 1.04 (+)	90.57 ± 0.70 (+)	90.12 ± 1.24 (+)	89.82 ± 0.93 (+)	90.12 ± 1.02 (+)
Glioma	**89.15 ± 0.37**	87.84 ± 0.50 (+)	88.56 ± 0.84 (+)	87.49 ± 0.83 (+)	86.65 ± 0.92 (+)	87.37 ± 0.82 (+)
Mesothelioma	**76.54 ± 1.55**	75.30 ± 2.12 (+)	75.30 ± 2.12 (+)	74.08 ± 2.03 (+)	73.77 ± 1.50 (+)	74.08 ± 2.02 (+)
W/T/L		8/1/0	6/3/0	8/1/0	9/0/0	9/0/0

The best values are in bold.

**Table 10:** Summary of the t test results for $f$ and the different metrics with LR.

	GA	GWO	PSO	WOA	FPA
Objective function* f*	9/0/0	9/0/0	9/0/0	9/0/0	9/0/0
Metrics on test sets					
ACC	8/1/0	6/3/0	8/1/0	9/0/0	9/0/0
AUC	7/2/0	5/4/0	8/1/0	9/0/0	9/0/0
Gmean	6/3/0	5/4/0	6/3/0	7/2/0	7/2/0
F1	7/2/0	5/4/0	8/1/0	7/2/0	7/2/0
Total (W/T/L)	28/8/0	21/15/0	30/6/0	32/4/0	32/4/0

The best values are in bold.

As can be observed from Table 9, our method obtained the best mean results in all databases. The GWO method obtained the same mean result only on the *SPECTF Heart* database. Moreover, in most cases (40 out of the 45 cases), these differences were significant. Specifically, in the t tests against the GWO method, our method “tied” on 3 databases and “won” on the other 6. As in the case of DA, it seems that the results obtained by GWO were relatively similar to those obtained by our MST.

From Table 10, it can be observed that our method obtained better results than the rest of the methods. Regarding the objective function $f$, all differences were significant in favor of our method. With respect to the metrics, compared to WOA and FPA, there were 32 significant differences in favor of MST; compared to PSO, there were 30 significant differences in favor of MST; compared to GA, there were 28 significant differences; and compared to GWO, there were 21 significant differences. As in the case of DA, there were no significant differences in favor of any of the other methods.

#### Results of SVM analysis

Finally, we analyze the results obtained for SVM. As in the case of LR, Table 11 shows the results of the $ACC$ metric, and Table 12 presents the summary of the t tests in terms of both the objective function $f$ and each of the metrics (Table 12).

**Table 11:** Results of $ACC$ (%) using SVM.

	MST	GA	GWO	PSO	WOA	FPA
Parkinson	**86.13 ± 3.49**	85.13 ± 1.58 (=)	85.13 ± 1.58 (=)	85.13 ± 1.58 (=)	84.61 ± 0.42 (+)	85.13 ± 1.58 (=)
QADC	**82.23 ± 1.07**	79.78 ± 2.84 (+)	81.88 ± 1.39 (=)	79.78 ± 2.84 (+)	78.04 ± 1.74 (+)	80.12 ± 2.50 (+)
SPECTF heart	**86.52 ± 1.86**	85.40 ± 1.10 (+)	85.78 ± 1.42 (=)	83.90 ± 3.03 (+)	84.63 ± 1.38 (+)	84.27 ± 2.30 (+)
WDBC	**97.53 ± 1.50**	96.13 ± 1.12 (+)	96.31 ± 1.55 (+)	96.31 ± 1.55 (+)	95.43 ± 1.23 (+)	95.60 ± 1.25 (+)
WPBC	**86.39 ± 3.26**	83.37 ± 3.23 (+)	84.37 ± 3.60 (+)	84.37 ± 3.60 (+)	82.34 ± 2.49 (+)	82.87 ± 3.34 (+)
Alzheimer	**80.88 ± 1.64**	78.36 ± 1.93 (+)	79.65 ± 1.78 (+)	78.10 ± 2.17 (+)	76.42 ± 2.26 (+)	77.20 ± 2.08 (+)
Cervical cancer	**90.27 ± 0.76**	89.82 ± 1.15 (+)	90.12 ± 1.02 (=)	89.37 ± 1.08 (+)	88.93 ± 1.41 (+)	89.82 ± 1.15 (+)
Glioma	**87.37 ± 1.50**	86.30 ± 1.13 (+)	86.41 ± 1.60 (+)	85.82 ± 1.17 (+)	86.18 ± 1.12 (+)	86.30 ± 1.13 (+)
Mesothelioma	**72.52 ± 3.75**	71.29 ± 2.14 (=)	70.98 ± 2.13 (+)	70.68 ± 1.50 (+)	70.37 ± 2.57 (+)	70.68 ± 1.50 (+)
W/T/L		7/2/0	5/4/0	8/1/0	9/0/0	8/1/0

The best values are in bold.

**Table 12:** Summary of the t test for $f$ and the different metrics with SVM.

	GA	GWO	PSO	WOA	FPA
Objective function* f*	9/0/0	9/0/0	9/0/0	9/0/0	9/0/0
Metrics on test sets					
ACC	7/2/0	5/4/0	8/1/0	9/0/0	8/1/0
AUC	7/2/0	6/3/0	8/1/0	9/0/0	8/1/0
Gmean	6/3/0	6/3/0	7/2/0	8/1/0	7/2/0
F1	6/3/0	5/4/0	7/2/0	8/1/0	8/1/0
Total (W/T/L)	26/10/0	22/14/0	30/6/0	34/2/0	31/5/0

Table 11 shows that, in all the databases, our method obtained better mean results than any of the baseline methods. Moreover, in most (37 out of the 45 cases), these differences were significant. Specifically, as in the cases of LR, in the t tests compared to GWO, our method “tied” in 4 databases and “won” in the other 5.

As can be observed in Table 12, our method obtained better results than any of the other methods. Regarding the objective function $f$, all the differences were significant in favor of our method. With respect to the metrics, compared to the WOA, there were 34 significant differences in favor of MST; compared to FPA, there were 31 significant differences in favor of MST; compared to PSO, there were 30 significant differences; compared to GA, there were 26 significant differences; and compared to GWO, there were 22 significant differences. As in the cases of DA and LR, there were no significant differences in favor of any of the other methods.

#### Comments on the set of results

Ultimately, the obtained results indicate the following:

- Considering all the databases, our method obtained better results than any of the other classifiers in terms of the objective function $f$ used in the variable selection process. Furthermore, with respect to the other methods, these differences were significant in all cases.
- In terms of all the metrics considered, the models obtained by our method in this process achieved better global results on the test set than the models obtained by any of the other methods. In fact, our method yielded the best mean results in most of the specific cases (that is, considering all the metrics, all the databases and all the classifiers used). Similar mean results were obtained in a very small number of cases, and there was one isolated case of a difference in favor of another method, although that difference was not significant.
- In addition, these differences in the metrics in favor of our method were significant in most of the cases. Thus, of the 108 tests that were conducted with each of the baseline methods (considering 4 metrics, 9 databases, and 3 classifiers), the differences in favor of our method were significant in 81 cases compared to GA, in 56 cases compared to GWO, in 89 cases compared to PSO, in 99 cases compared to WOA, and in 94 cases compared to FPA. In the rest of the cases, there were no significant differences in favor of any method. Therefore, it seems that only the results of the GWO method were similar to those of MST, and only in half of the cases.

## Conclusions

The use of classification models is increasing in the field of medicine, since they help to improve the diagnosis of diseases. Advances in these models are necessary and useful to increase the precision and reduce the uncertainty of diagnoses. Currently, there are databases with several features that aid in the creation of models. However, not all features contribute to this task in the same way. Some features may be irrelevant or noisy, and they can deteriorate the performance of the models. There may also be negative interactions among features, which are not always easy to distinguish and can also reduce the performance of the models. Thus, feature selection is critical in the field of medicine: it improves the diagnosis of diseases, identifies which features lead to a better diagnosis, and identifies which risk factors (personal traits, habits, etc.) have the largest impacts on the disease. From a computational perspective, feature selection is a hard problem due to the complexity of the search space. The most commonly used feature selection methods are based on evolutionary strategies, such as GAs, swarm optimization techniques, and bioinspired algorithms. This study proposes a wrapper method that creates solutions and improves them through a tabu search process in a multistart framework. Therefore, the strategy it follows is not among the ones that are most frequently used in this field. The results of different computational tests in medical databases show that our method outperforms other recently developed wrapper methods in the literature. The tests were conducted using different classifiers and metrics, and the results are accompanied by statistical tests that strengthen the conclusions.

In general, the main limitation of our method is that it requires significant adaptation to each specific problem and costs a great deal of effort to implement. Other recent methods, such as evolutionary methods, seem to be more intuitive and easier to implement. On the other hand, our method demonstrates better performance considering different metrics, databases, statistical tests, etc.

In short, the contribution of this work is the development of a method that, on the one hand, is based on different strategies than those used in recent methods, and on the other hand, significantly improves the performance of these methods.

## Data Availability

The datasets used for the current study are available in the following links/repositories: https://archive.ics.uci.edu/, https://www.ubu.es/metaheuristicos-grinubumet/ejemplos-y-datos-de-problemas. The code is available on the following GitHub link: https://github.com/japachecob/MSTabuFeatureSelection.
